# Underlying Medical Conditions Associated With Severe COVID-19 Illness Among Children

**DOI:** 10.1001/jamanetworkopen.2021.11182

**Published:** 2021-06-07

**Authors:** Lyudmyla Kompaniyets, Nickolas T. Agathis, Jennifer M. Nelson, Leigh Ellyn Preston, Jean Y. Ko, Brook Belay, Audrey F. Pennington, Melissa L. Danielson, Carla L. DeSisto, Jennifer R. Chevinsky, Lyna Z. Schieber, Hussain Yusuf, James Baggs, William R. Mac Kenzie, Karen K. Wong, Tegan K. Boehmer, Adi V. Gundlapalli, Alyson B. Goodman

**Affiliations:** 1 COVID-19 Response, US Centers for Disease Control and Prevention, Atlanta, Georgia; 2 Epidemic Intelligence Service, Center for Surveillance, Epidemiology and Laboratory Services, US Centers for Disease Control and Prevention, Atlanta, Georgia; 3 US Public Health Service Commissioned Corps, Rockville, Maryland

## Abstract

**Importance:**

Information on underlying conditions and severe COVID-19 illness among children is limited.

**Objective:**

To examine the risk of severe COVID-19 illness among children associated with underlying medical conditions and medical complexity.

**Design, Setting, and Participants:**

This cross-sectional study included patients aged 18 years and younger with *International Statistical Classification of Diseases, Tenth Revision, Clinical Modification *code U07.1 (COVID-19) or B97.29 (other coronavirus) during an emergency department or inpatient encounter from March 2020 through January 2021. Data were collected from the Premier Healthcare Database Special COVID-19 Release, which included data from more than 800 US hospitals. Multivariable generalized linear models, controlling for patient and hospital characteristics, were used to estimate adjusted risk of severe COVID-19 illness associated with underlying medical conditions and medical complexity.

**Exposures:**

Underlying medical conditions and medical complexity (ie, presence of complex or noncomplex chronic disease).

**Main Outcomes and Measures:**

Hospitalization and severe illness when hospitalized (ie, combined outcome of intensive care unit admission, invasive mechanical ventilation, or death).

**Results:**

Among 43 465 patients with COVID-19 aged 18 years or younger, the median (interquartile range) age was 12 (4-16) years, 22 943 (52.8%) were female patients, and 12 491 (28.7%) had underlying medical conditions. The most common diagnosed conditions were asthma (4416 [10.2%]), neurodevelopmental disorders (1690 [3.9%]), anxiety and fear-related disorders (1374 [3.2%]), depressive disorders (1209 [2.8%]), and obesity (1071 [2.5%]). The strongest risk factors for hospitalization were type 1 diabetes (adjusted risk ratio [aRR], 4.60; 95% CI, 3.91-5.42) and obesity (aRR, 3.07; 95% CI, 2.66-3.54), and the strongest risk factors for severe COVID-19 illness were type 1 diabetes (aRR, 2.38; 95% CI, 2.06-2.76) and cardiac and circulatory congenital anomalies (aRR, 1.72; 95% CI, 1.48-1.99). Prematurity was a risk factor for severe COVID-19 illness among children younger than 2 years (aRR, 1.83; 95% CI, 1.47-2.29). Chronic and complex chronic disease were risk factors for hospitalization, with aRRs of 2.91 (95% CI, 2.63-3.23) and 7.86 (95% CI, 6.91-8.95), respectively, as well as for severe COVID-19 illness, with aRRs of 1.95 (95% CI, 1.69-2.26) and 2.86 (95% CI, 2.47-3.32), respectively.

**Conclusions and Relevance:**

This cross-sectional study found a higher risk of severe COVID-19 illness among children with medical complexity and certain underlying conditions, such as type 1 diabetes, cardiac and circulatory congenital anomalies, and obesity. Health care practitioners could consider the potential need for close observation and cautious clinical management of children with these conditions and COVID-19.

## Key Points

**Question:**

Among children with a COVID-19 diagnosis, what conditions are common, and which are associated with severe COVID-19 illness?

**Findings:**

In this cross-sectional study of 43 465 patients aged 18 years or younger with COVID-19, more than one-quarter had 1 or more underlying condition; asthma, obesity, neurodevelopmental disorders, and certain mental health conditions were most common. Certain conditions as well as medical complexity were associated with a higher risk of severe COVID-19 illness.

**Meaning:**

These findings expand the knowledge available regarding children with COVID-19 and could inform pediatric clinical practice and public health priorities, such as prevention and mitigation of COVID-19.

## Teaser

This cross-sectional study examines the risk of severe COVID-19 illness among children associated with underlying medical conditions and medical complexity.

## Introduction

Our understanding of COVID-19 illness in children is limited.^1^ To date, most children with SARS-CoV-2 infection have been asymptomatic or had mild COVID-19 symptoms, but some children have had severe illness.^2^ Prior literature identified risk factors for severe COVID-19 illness in children, including being younger than 1 year or having an underlying medical condition, such as congenital heart disease, asthma, obesity, diabetes, or neurologic conditions.^2,3,4,5,6,7^ Approximately 1 in 4 US children have a chronic condition, including asthma, obesity, and behavioral or learning disorders.^8^

Previous studies on risk factors among children were limited by small sample sizes that affected the study’s ability to detect statistically significant associations and by a limited ability to follow up children over multiple encounters.^2,3^ By using a large electronic administrative health care data set, we sought to describe common underlying medical conditions and medical complexity as well as their associations with the risk of hospitalization or severe illness among children seeking care in the hospital.

## Methods

This activity was reviewed by the US Centers for Disease Control and Prevention and was deemed exempt from institutional review board oversight per 45 CFR §46.101(b)(4) and exempt from patient informed consent per 45 CFR §164.506(d)(2)(ii)(B) because the disclosed Premier Healthcare Database Special COVID-19 Release (PHD-SR) data are considered deidentified. This report was guided by and conforms to the Strengthening the Reporting of Observational Studies in Epidemiology (STROBE) reporting guideline for cross-sectional studies.^9^

### Data Source

This analysis used the PHD-SR (release date, March 15, 2021), a large, hospital-based, all-payer database.^10^ Among more than 900 geographically dispersed US hospitals in PHD-SR, 872 contributed both emergency department (ED) and inpatient encounters to PHD-SR and were selected for this study.

### Study Population

The sample included patients aged 18 years or younger who had an inpatient or ED encounter with a primary or secondary COVID-19 discharge diagnosis from March 1, 2020, through January 31, 2021 (eFigure 1 in the Supplement). The *International Classification of Diseases, Tenth Revision, Clinical Modification* (*ICD-10-CM*) diagnosis code U07.1 (COVID-19, virus identified) was used from April 1, 2020, through January 31, 2021, and code B97.29 (other coronavirus as the cause of diseases classified elsewhere) was used from March 1 through April 30, 2020.^11^ Patients with missing sex (n = 56) were omitted from the analysis. Patient characteristics, including race and ethnicity categories, were provided in the information from patient medical records in the PHD-SR. Categories were defined separately for race and ethnicity by PHD-SR and were combined into a single variable (race/ethnicity) for this study. Given the known racial and ethnic disparities in incidence and severity of both COVID-19 and several of the underlying conditions analyzed, race/ethnicity is a key confounder to consider.^12^

### Measures and Outcomes

Two outcomes were considered: (1) hospitalization and (2) severe illness when hospitalized, a single severity indicator for experiencing an intensive care unit (ICU) or stepdown unit admission, invasive mechanical ventilation (IMV), or death. Hospitalization was defined by having an inpatient encounter, death was defined using patient discharge status, and ICU admission and IMV were determined using patient billing records.

This study assessed 2 exposures of interest: (1) specific underlying medical conditions and (2) medical complexity (defined later in this section). Both exposures were captured using *ICD-10-CM* diagnosis codes from all inpatient and hospital-based outpatient medical records in PHD-SR from January 1, 2019, up to and including a patient’s initial COVID-19 encounter (henceforth, measurement period). Three *ICD-10-CM* codes related to dependence on oxygen or ventilator or having received tracheostomy were excluded during the patient’s COVID-19 encounter, as they could be a part of COVID-19 treatment.

A multistep approach was used to identify underlying medical conditions (eFigure 2 in the Supplement). First, we used the Chronic Condition Indicator (CCI) to identify chronic *ICD-10-CM* codes (11 803 of 73 205 total *ICD-10-CM* codes). Second, we used Clinical Classifications Software Refined (CCSR) to aggregate these codes into 316 meaningful categories.^13,14^ The CCI included some conditions that could be acute complications of COVID-19. To further differentiate and isolate truly underlying conditions from acute sequelae of COVID-19, a panel of physicians (N.T.A., J.M.N., B.B., W.R.M., K.K.W., and panel member H.G.R.) classified the 316 CCSR categories as (1) likely underlying conditions that potentially preceded the acute COVID-19 illness (275 categories); (2) likely acute conditions that were likely the result of acute COVID-19 illness (13 categories); or (3) indeterminate conditions that could be either acute or underlying or both (28 categories). We used the likely underlying CCSR categories for our analysis of underlying medical conditions and excluded the likely acute and indeterminate categories. Individuals with codes for both type 1 and type 2 diabetes were classified as having type 1 diabetes if 50% or more of their encounters included diagnosis codes for type 1 diabetes.^15^

Medical complexity was defined using the validated pediatric medical complexity algorithm (PMCA) as presence of complex chronic disease (C-CD), presence of noncomplex chronic disease (NC-CD), and absence of chronic disease (reference category).^16^ Presence of C-CD was defined as having at least 1 encounter with a progressive condition, at least 1 encounter with malignant neoplasms, or at least 1 encounter per body system for 2 different body systems during the measurement period. Presence of NC-CD was defined as having at least 1 encounter for a single body system not flagged as progressive. Absence of chronic disease was defined as having none of the previously described encounters during the measurement period.^16^

### Statistical Analysis

We used frequencies and percentages to describe the characteristics of the patient sample and hospitals they visited. We then listed the most frequently documented underlying medical conditions in the sample. We used multivariate generalized linear models with Poisson distribution, log link function, and robust standard errors to estimate the associations of frequent (ie, prevalence >0.7%) underlying medical conditions and medical complexity with the outcomes of hospitalization (reference group, patients seen in ED only) and severe illness among hospitalized patients (reference group, hospitalized patients without severe illness). Poisson distribution with robust standard errors was used instead of binomial distribution because of model convergence issues.^17,18^ All models used robust standard errors clustered on hospital identification^19^ and included frequent (ie, prevalence >0.7%) underlying medical conditions, age group, sex, race/ethnicity, payer type, hospital urbanicity, hospital US Census region, admission month, and admission month squared.

We also conducted stratified analyses of frequent conditions by age group (frequency ≥1% within each age group) and estimated their association with risk of hospitalization and severe COVID-19 illness. The stratified analysis of children aged 1 year or younger included prematurity (gestational age <37 weeks at birth; *ICD-10-CM* codes, P07.2X and P07.3X) as an additional covariate and a potential risk factor for severe illness.

Additionally, 2 sensitivity analyses were performed, using 316 CCSR categories without excluding those marked as indeterminate and likely acute by clinical review. One sensitivity analysis was performed in a full sample, another was performed with the subset of patients with at least 1 encounter before their first COVID-19 encounter. *P* < .05 was considered statistically significant, and all tests were 2-tailed. All analyses were conducted using R version 4.0.2 (R Project for Statistical Computing) and Stata version 15.1 (StataCorp).

## Results

Among 3 782 157 children with ED or inpatient encounters in the PHD-SR, 43 465 (1.1%) met inclusion criteria as patients with COVID-19 (Table 1). Of children with COVID-19, 22 943 (52.8%) were female patients, 14 333 (33.0%) were Hispanic or Latino individuals, and 10 488 (24.1%) were non-Hispanic Black individuals. The median (interquartile range) age was 12 (4-16) years. Among children with COVID-19, 4302 (9.9%) were hospitalized (ie, had an inpatient visit), of whom 1273 (29.6%) had an ICU admission, 277 (6.4%) received IMV, and 38 (0.9%) died.

**Table 1.** Characteristics of the Sample^a^

Characteristic^b^	Children, No. (%)					
	ED or inpatient encounter (N = 3 782 157)	With COVID-19				
		ED or inpatient encounter (n = 43 465)	ED encounter only (n = 39 163)	Inpatient encounter (n = 4302)	Inpatient encounter and no severe illness (n = 3013)	Inpatient encounter and severe illness (n = 1289)
With underlying medical conditions^c^	856 419 (22.6)	12 491 (28.7)	9783 (25.0)	2708 (62.9)	1702 (56.5)	1006 (78.0)
With chronic disease^d^	549 714 (14.5)	9524 (21.9)	7208 (18.4)	2316 (53.8)	1345 (44.6)	971 (75.3)
Noncomplex chronic	423 453 (11.2)	6450 (14.8)	5489 (14.0)	961 (22.3)	651 (21.6)	310 (24.0)
Complex chronic	126 261 (3.3)	3074 (7.1)	1719 (4.4)	1355 (31.5)	694 (23.0)	661 (51.3)
Sex						
Female	1 881 952 (49.8)	22 943 (52.8)	20 615 (52.6)	2328 (54.1)	1735 (57.6)	593 (46.0)
Male	1 900 205 (50.2)	20 522 (47.2)	18 548 (47.4)	1974 (45.9)	1278 (42.4)	696 (54.0)
Age, y						
Median (IQR)	5 (0-13)	12 (4-16)	12 (4-16)	12 (1-17)	12 (1-17)	13 (3-17)
<1	1 115 079 (29.5)	5450 (12.5)	4501 (11.5)	949 (22.1)	750 (24.9)	199 (15.4)
1	238 472 (6.3)	2454 (5.6)	2260 (5.8)	194 (4.5)	141 (4.7)	53 (4.1)
2-5	630 996 (16.7)	5034 (11.6)	4615 (11.8)	419 (9.7)	257 (8.5)	162 (12.6)
6-11	670 919 (17.7)	7552 (17.4)	7056 (18.0)	496 (11.5)	306 (10.2)	190 (14.7)
12-18	1 126 691 (29.8)	22 975 (52.9)	20 731 (52.9)	2244 (52.2)	1559 (51.7)	685 (53.1)
Race/ethnicity						
Hispanic or Latino	752 864 (19.9)	14 333 (33.0)	12 884 (32.9)	1449 (33.7)	1062 (35.2)	387 (30.0)
Non-Hispanic White	1 771 049 (46.8)	13 229 (30.4)	11 781 (30.1)	1448 (33.7)	1017 (33.8)	431 (33.4)
Non-Hispanic Black	701 639 (18.6)	10 488 (24.1)	9693 (24.8)	795 (18.5)	506 (16.8)	289 (22.4)
Non-Hispanic Asian	87 965 (2.3)	858 (2.0)	760 (1.9)	98 (2.3)	68 (2.3)	30 (2.3)
Non-Hispanic other race	289 888 (7.7)	3179 (7.3)	2860 (7.3)	319 (7.4)	216 (7.2)	103 (8.0)
Unknown	178 752 (4.7)	1378 (3.2)	1185 (3.0)	193 (4.5)	144 (4.8)	49 (3.8)
Payer type						
Private	1 213 599 (32.1)	10 094 (23.2)	8997 (23.0)	1097 (25.5)	752 (25.0)	345 (26.8)
Public, ie, Medicare/Medicaid	2 175 991 (57.5)	29 479 (67.8)	26 586 (67.9)	2893 (67.2)	2050 (68.1)	843 (65.4)
Charity care, indigent, or self-pay	221 390 (5.9)	1722 (4.0)	1604 (4.1)	118 (2.7)	77 (2.6)	41 (3.2)
Payer unknown	171 177 (4.5)	2170 (5.0)	1976 (5.0)	194 (4.5)	134 (4.4)	60 (4.7)
Severity markers						
Hospitalization	1 037 653 (27.4)	4302 (9.9)	0	4302 (100.0)	3013 (100.0)	1289 (100.0)
ICU admission	121 482 (3.2)	1273 (2.9)	0	1273 (29.6)	0	1273 (98.8)
Invasive mechanical ventilation	33 083 (0.9)	289 (0.7)	12 (0.3)	277 (6.4)	0	277 (21.5)
Death	5145 (0.1)	NA	NA	38 (0.9)	0	38 (2.9)
Hospital characteristics						
Urbanicity						
Rural	640 976 (16.9)	6455 (14.9)	6052 (15.5)	403 (9.4)	257 (8.5)	146 (11.3)
Urban	3 141 181 (83.1)	37 010 (85.1)	33 111 (84.5)	3899 (90.6)	2756 (91.5)	1143 (88.7)
US census region						
Midwest	785 123 (20.8)	7092 (16.3)	6376 (16.3)	716 (16.6)	522 (17.3)	194 (15.1)
Northeast	532 627 (14.1)	5426 (12.5)	4670 (11.9)	756 (17.6)	547 (18.2)	209 (16.2)
South	1 817 933 (48.1)	24 880 (57.2)	22 570 (57.6)	2310 (53.7)	1568 (52.0)	742 (57.6)
West	646 474 (17.1)	6067 (14.0)	5547 (14.2)	520 (12.1)	376 (12.5)	144 (11.2)

Abbreviations: ED, emergency department; ICU, intensive care unit; IQR, interquartile range.

^a^ The sample was defined as children aged 18 years or younger with a COVID-19 diagnosis during an ED or inpatient encounter in hospitals that reported both ED and inpatient encounters to Premier Healthcare Database Special COVID-19 Release, March 2020 through January 2021.

^b^ Percentages in some categories may not add up to 100%, due to being not mutually exclusive, rounding or missing/unknown values, or cell size suppression (cells with <10 patients suppressed).

^c^ Underlying medical conditions included only likely underlying categories in the Clinical Classifications Software Refined, as defined in the Methods.

^d^ Chronic disease was defined by the Pediatric Medical Complexity Algorithm.

Underlying medical conditions were present in 12 491 children with COVID-19 (28.7%) and 2708 hospitalized children with COVID-19 (62.9%) (Table 1). Condition frequencies stratified by care setting are in eTable 1 of the Supplement. As defined by PMCA, chronic disease, including NC-CD and C-CD, was present in 9524 children (21.9%) and 2316 hospitalized children (53.8%) (Table 1).

**Figure 1.**
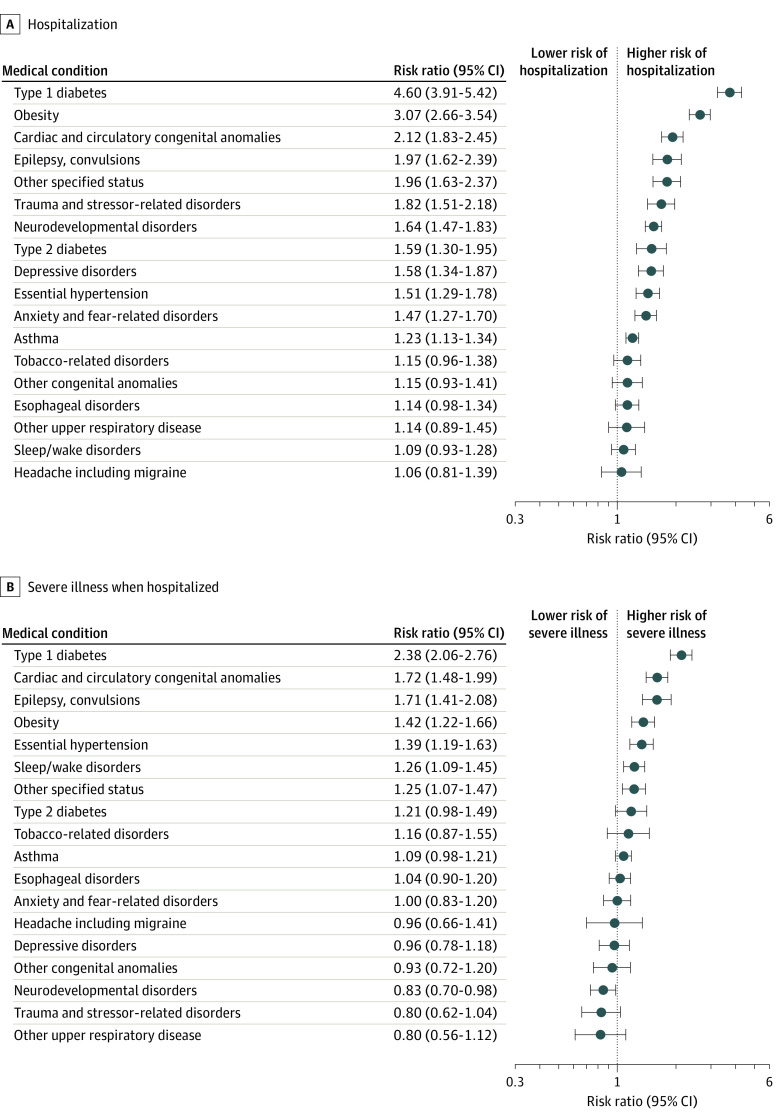
Association Between Underlying Medical Conditions and Risk of Hospitalization or Severe Illness When Hospitalized in the Sample Underlying medical conditions were defined as described n the Methods section. The sample was defined as children aged 18 years or younger with a COVID-19 diagnosis during an emergency department or inpatient encounter in hospitals that reported both emergency department and inpatient encounters to Premier Healthcare Database Special COVID-19 Release, March 2020 through January 2021. Each panel represents results of a single generalized linear model with Poisson distribution and log link function, that includes the following covariates: frequent (ie, prevalence >0.7%) underlying medical conditions, age group, sex, race/ethnicity, payer type, hospital urbanicity, hospital US Census region, admission month, and admission month squared. The reference group for each underlying condition was absence of that condition; the reference group for type 1 and type 2 diabetes was no diabetes.

We identified 18 underlying medical conditions with frequency greater than 0.7% in the sample, with the most common being asthma (4416 [10.2%]), neurodevelopmental disorders (1690 [3.9%]), anxiety and fear-related disorders (1374 [3.2%]), depressive disorders (1209 [2.8%]), and diagnosed obesity (1071 [2.5%]) (Table 2). Of these conditions, 12 were associated with at least 1 of the 2 severe COVID-19 outcomes. Type 1 diabetes, obesity, and cardiac and circulatory congenital anomalies were the strongest risk factors for hospitalization, with adjusted risk ratios (aRRs) of 4.60 (95% CI, 3.91-5.42), 3.07 (95% CI, 2.66-3.54), and 2.12 (95% CI, 1.83–2.45), respectively. Other conditions associated with a higher risk of hospitalization included epilepsy and/or convulsions (aRR, 1.97; 95% CI, 1.62-2.39); other specified status, a category indicating dependence on medical support, such as gastrostomy status (aRR, 1.96; 95% CI, 1.63-2.37); trauma and stressor-related disorders (aRR, 1.82; 95% CI, 1.51-2.18); neurodevelopmental disorders (aRR, 1.64; 95% CI, 1.47-1.83); type 2 diabetes (aRR, 1.59; 95% CI, 1.30-1.95); depressive disorders (aRR, 1.58; 95% CI, 1.34-1.87); essential hypertension (aRR, 1.51; 95% CI, 1.29-1.78); anxiety and fear-related disorders (aRR, 1.47; 95% CI, 1.27-1.70); and asthma (aRR, 1.23; 95% CI, 1.13-1.34) (Figure 1).

**Table 2.** The Most Frequent Underlying Medical Conditions in the Sample^a^

CCSR category	No. (%)
Asthma	4416 (10.2)
Neurodevelopmental disorders	1690 (3.9)
Anxiety and fear-related disorders	1374 (3.2)
Depressive disorders	1209 (2.8)
Obesity	1071 (2.5)
Esophageal disorders	879 (2.0)
Tobacco-related disorders	686 (1.6)
Diabetes	
Any	627 (1.4)
Type 1 diabetes	307 (0.7)
Type 2 diabetes	320 (0.7)
Epilepsy or convulsions	559 (1.3)
Cardiac and circulatory congenital anomalies	510 (1.2)
Other specified and unspecified upper respiratory disease; top code, allergic rhinitis	498 (1.1)
Essential hypertension	436 (1.0)
Trauma and stressor-related disorders; top code, posttraumatic stress disorder	432 (1.0)
Other specified status; top code, gastrostomy status^b^	403 (0.9)
Other specified and unspecified congenital anomalies	393 (0.9)
Headache, including migraine	366 (0.8)
Sleep/wake disorders; top code, sleep apnea	329 (0.8)

Abbreviation: CCSR, Clinical Classifications Software Refined.

^a^ Underlying medical conditions included only likely underlying CCSR categories, as defined in the Methods. The sample was defined as children aged 18 years or younger with a COVID-19 diagnosis during an emergency department or inpatient encounter in hospitals that reported both emergency department and inpatient encounters to Premier Healthcare Database Special COVID-19 Release, March 2020 through January 2021.

^b^ Other specified status (CCSR category FAC025) includes children who required specific medical support, such as gastrostomy, tracheostomy, or renal dialysis, or were awaiting organ transplant.

Among 4302 children hospitalized with COVID-19, risk of severe COVID-19 illness (ie, experiencing ICU admission, IMV, or death) was the highest among children with type 1 diabetes (aRR, 2.38; 95% CI, 2.06-2.76), cardiac and circulatory congenital anomalies (aRR, 1.72; 95% CI, 1.48-1.99), and epilepsy and/or convulsions (aRR, 1.71; 95% CI, 1.41-2.08). Other conditions associated with higher risk were obesity (aRR, 1.42; 95% CI, 1.22-1.66); essential hypertension (aRR, 1.39; 95% CI, 1.19-1.63); sleep/wake disorders, including sleep apnea (aRR, 1.26; 95% CI, 1.09-1.45); and other specified status (aRR, 1.25; 95% CI, 1.07-1.47) (Figure 1).

Stratified analyses showed that among children aged 1 year or younger and 2 to 5 years, cardiac and congenital anomalies were both frequent (306 of 7904 [3.9%]; 84 of 5034 [1.7%]) and associated with a higher risk of severe illness when hospitalized (aRR, 1.89; 95% CI, 1.48-2.41; aRR, 1.50; 95% CI, 1.05-2.16) (Table 3). Among children aged 1 year or younger, prematurity was a frequent (479 [6.1%]) and significant risk factor for severe COVID-19 illness (aRR, 1.83; 95% CI, 1.47-2.29). Among hospitalized patients aged 12 to 18 years, risk factors for severe COVID-19 illness were type 1 diabetes (aRR, 2.47; 95% CI, 2.12-2.87), epilepsy and/or convulsions (aRR, 1.89; 95% CI, 1.53-2.34), obesity (aRR, 1.57; 95% CI, 1.32-1.85), essential hypertension (aRR, 1.23; 95% CI, 1.01-1.51), and asthma (aRR, 1.17; 95% CI, 1.02-1.33).

**Table 3.** Most Frequent Underlying Medical Conditions and Their Association With Risk of Hospitalization or Severe Illness When Hospitalized, Stratified by Age Group^a^

Medical condition	No. (%)		Risk ratio (95% CI)	
	Full sample	Inpatient subset	Full sample: hospitalization^b^	Inpatient subset: severe illness^b^^,^^c^
**Age, ≤1 y**				
No.	7904	1143	7904	1143
Prematurity	479 (6.1)	139 (12.2)	1.42 (1.17-1.72)^d^	1.83 (1.47-2.29)^d^
Cardiac and circulatory congenital anomalies	306 (3.9)	136 (11.9)	2.00 (1.67-2.38)^d^	1.89 (1.48-2.41)^d^
Other congenital anomalies	299 (3.8)	51 (4.5)	0.92 (0.69-1.22)	1.12 (0.72-1.73)
Esophageal disorders	262 (3.3)	77 (6.7)	1.18 (0.93-1.50)	1.19 (0.86-1.65)
Musculoskeletal congenital conditions	142 (1.8)	43 (3.8)	1.03 (0.81-1.32)	1.33 (0.92-1.92)
Genitourinary congenital anomalies	141 (1.8)	45 (3.9)	1.58 (1.20-2.08)^d^	0.97 (0.63-1.48)
Digestive congenital anomalies	132 (1.7)	41 (3.6)	1.18 (0.91-1.52)	0.52 (0.28-0.96)^d^
Asthma	123 (1.6)	31 (2.7)	1.34 (1.00-1.80)^d^	1.39 (0.88-2.19)
Other specified status^e^	92 (1.2)	56 (4.9)	1.62 (1.21-2.17)^d^	1.48 (0.98-2.22)
**Age, 2-5 y**				
No.	5034	419	5034	419
Asthma	449 (8.9)	89 (21.2)	1.88 (1.43-2.48)^d^	1.04 (0.73-1.50)
Neurodevelopmental disorders	195 (3.9)	63 (15.0)	1.88 (1.31-2.70)^d^	0.61 (0.43-0.88)^d^
Esophageal disorders	115 (2.3)	43 (10.3)	0.97 (0.65-1.46)	0.88 (0.59-1.31)
Epilepsy and/or convulsions	111 (2.2)	45 (10.7)	2.28 (1.66-3.15)^d^	1.95 (1.40-2.74)^d^
Other specified status^e^	89 (1.8)	47 (11.2)	1.40 (0.85-2.32)	1.15 (0.80-1.66)
Cardiac and circulatory congenital anomalies	84 (1.7)	39 (9.3)	2.26 (1.46-3.49)^d^	1.50 (1.05-2.16)^d^
Implant device or graft-related encounter	77 (1.5)	33 (7.9)	1.92 (1.31-2.81)^d^	0.86 (0.56-1.32)
Other upper respiratory disease	71 (1.4)	15 (3.6)	1.68 (1.04-2.73)^d^	0.59 (0.25-1.43)
Sleep/wake disorders	56 (1.1)	24 (5.7)	0.77 (0.50-1.17)	1.50 (0.82-2.77)
Musculoskeletal congenital conditions	52 (1.0)	27 (6.4)	1.38 (0.91-2.10)	1.00 (0.61-1.63)
**Age, 6-11 y**				
No.	7552	496	7552	496
Asthma	995 (13.2)	114 (23.0)	1.40 (1.15-1.71)^d^	0.97 (0.77-1.22)
Neurodevelopmental disorders	390 (5.2)	91 (18.3)	2.20 (1.64-2.95)^d^	0.75 (0.54-1.05)
Obesity	120 (1.6)	41 (8.3)	3.65 (2.62-5.10)^d^	1.17 (0.82-1.66)
Other upper respiratory disease	112 (1.5)	17 (3.4)	1.52 (1.00-2.32)	0.98 (0.70-1.37)
Epilepsy and/or convulsions	110 (1.5)	45 (9.1)	2.09 (1.35-3.23)^d^	1.54 (1.05-2.26)^d^
Esophageal disorders	104 (1.4)	29 (5.8)	1.04 (0.68-1.60)	1.12 (0.72-1.75)
Other specified status^e^	83 (1.1)	45 (9.1)	1.69 (1.09-2.63)^d^	0.98 (0.63-1.52)
Anxiety and fear-related disorders	82 (1.1)	32 (6.5)	2.27 (1.35-3.83)^d^	1.13 (0.74-1.74)
Sleep/wake disorders	80 (1.1)	35 (7.1)	1.78 (1.21-2.62)^d^	1.82 (1.35-2.45)^d^
**Age, 12-18 y**				
No.	22 975	2244	22 975	2244
Asthma	2849 (12.4)	373 (16.6)	1.06 (0.96-1.18)	1.17 (1.02-1.33)^d^
Anxiety and fear-related disorders	1279 (5.6)	335 (14.9)	1.44 (1.25-1.65)^d^	0.95 (0.79-1.16)
Depressive disorders	1153 (5.0)	333 (14.8)	1.64 (1.39-1.94)^d^	0.95 (0.77-1.16)
Neurodevelopmental disorders	1064 (4.6)	275 (12.3)	1.71 (1.52-1.92)^d^	0.94 (0.77-1.14)
Obesity	916 (4.0)	384 (17.1)	3.24 (2.83-3.71)^d^	1.57 (1.32-1.85)^d^
Tobacco-related disorders	678 (3.0)	106 (4.7)	1.18 (0.98-1.42)	1.17 (0.86-1.58)
Esophageal disorders	398 (1.7)	118 (5.3)	1.49 (1.19-1.86)^d^	1.19 (0.97-1.47)
Trauma and stressor-related disorders	377 (1.6)	162 (7.2)	1.73 (1.42-2.11)^d^	0.87 (0.63-1.20)
Essential hypertension	319 (1.4)	127 (5.7)	1.29 (1.05-1.59)^d^	1.23 (1.01-1.51)^d^
Menstrual disorders	315 (1.4)	56 (2.5)	1.14 (0.87-1.49)	1.09 (0.72-1.67)
Headache, including migraine	311 (1.4)	56 (2.5)	0.92 (0.68-1.24)	0.86 (0.56-1.34)
Type 2 diabetes	289 (1.3)	97 (4.3)	1.60 (1.29-1.99)^d^	1.23 (0.98-1.53)
Other upper respiratory disease	285 (1.2)	47 (2.1)	1.03 (0.75-1.39)	0.69 (0.40-1.21)
Epilepsy and/or convulsions	285 (1.2)	117 (5.2)	2.52 (2.07-3.07)^d^	1.89 (1.53-2.34)^d^
Type 1 diabetes	255 (1.1)	126 (5.6)	4.33 (3.60-5.20)^d^	2.47 (2.12-2.87)^d^
Bipolar and related disorders	257 (1.1)	67 (3.0)	1.09 (0.84-1.42)	0.79 (0.49-1.26)

^a^ Underlying medical conditions included only likely underlying categories, as defined in the Methods. The sample was defined as children aged 18 years or younger with a COVID-19 diagnosis during an emergency department or inpatient encounter in hospitals that report both emergency department and inpatient encounters to Premier Healthcare Database, March 2020 through January 2021.

^b^ Each column represents the results of a single generalized linear model with Poisson distribution and log link function, stratified by age group (≤1, 2-5, 6-11, 12-18 years) that includes frequent (prevalence >1%) underlying medical conditions, sex, race/ethnicity, payer type, hospital urbanicity, hospital US Census region, admission month, and admission month squared. Prematurity (gestational age <37 weeks at birth, *International Statistical Classification of Diseases, Tenth Revision, Clinical Modification *codes P07.2X and P07.3X) was included as an additional covariate for children aged 1 year or younger. The reference group for each underlying condition was the absence of that condition; the reference group for type 1 and type 2 diabetes was no diabetes.

^c^ Severe illness among hospitalized patients includes intensive care unit admission, invasive mechanical ventilation, or death.

^d^ *P* < .05.

^e^ Other specified status included children who required specific medical support, such as gastrostomy, tracheostomy, or renal dialysis, or were awaiting organ transplant.

**Figure 2.**
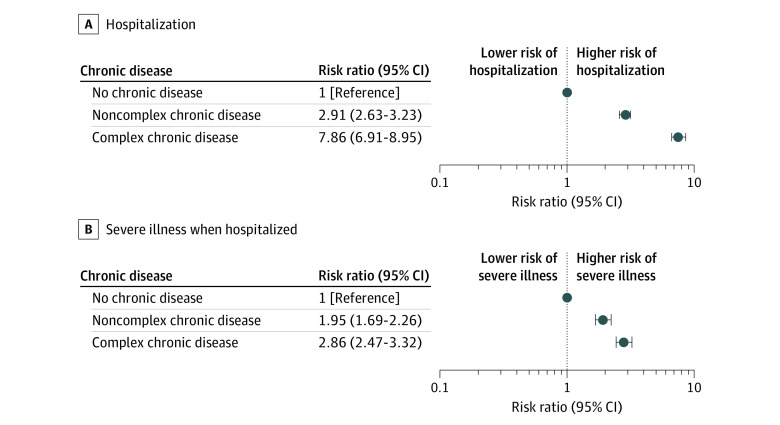
Association Between Medical Complexity and Risk of Hospitalization or Severe Illness When Hospitalized in the Sample Medical complexity was defined by the Pediatric Medical Complexity Algorithm. The sample was defined as children aged 18 years or younger with a COVID-19 diagnosis during an emergency department or inpatient encounter in hospitals that reported both emergency department and inpatient encounters to Premier Healthcare Database Special COVID-19 Release, March 2020 through January 2021. Each panel represents results of a single generalized linear model with Poisson distribution and log link function, that includes the following covariates: medical complexity (presence of noncomplex chronic disease, presence of complex chronic disease, and no chronic disease as reference group), age group, sex, race/ethnicity, payer type, hospital urbanicity, hospital US Census region, admission month, and admission month squared.

Medical complexity was associated with risk of hospitalization and severe illness when hospitalized (Figure 2). Compared with children without chronic disease, children with NC-CD and C-CD were 2.91 times (95% CI, 2.63-3.23) and 7.86 times (95% CI, 6.91-8.95) more likely to be hospitalized, respectively. Compared with hospitalized children without chronic disease, hospitalized children with NC-CD and C-CD were 1.95 times (95% CI, 1.69-2.26) and 2.86 times (95% CI, 2.47-3.32) more likely to have severe illness, respectively.

The first sensitivity analysis, performed without excluding likely acute and indeterminate CCSR categories, found 3 frequent (>0.7%) indeterminate categories that were associated with at least 1 outcome of severe illness: coagulation and hemorrhagic disorders, diseases of white blood cells, and other nutritional and metabolic disorders (eFigure 3 in the Supplement). The risk estimates of most previously found underlying medical conditions were slightly lower with the inclusion of these 3 conditions in regression models.

The second sensitivity analysis was performed using CCSR categories captured prior to the first COVID-19 visit in a subset of 20 773 children (eTable 2 and eTable 3 in the Supplement). Risk estimates of certain conditions were higher (eg, type 2 diabetes) or lower (eg, obesity) in magnitude compared with our main findings (eFigure 4 in the Supplement).

## Discussion

In this cross-sectional study of 43 465 US children with a COVID-19 diagnosis in ED or inpatient encounters from March 2020 through January 2021, the most commonly documented underlying conditions were asthma, neurodevelopmental disorders, anxiety and fear-related disorders, depressive disorders, and obesity. Children with cardiac and circulatory congenital anomalies, essential hypertension, and type 1 diabetes had higher risk of both hospitalization and severe illness when hospitalized. Limited reports of small case series or cohort studies suggest that children with congenital heart disease might be at increased risk of severe COVID-19 illness.^6,20^ A nationwide study of adults and children in England found type 1 diabetes to be associated with higher odds of in-hospital death from COVID-19.^21^ Our study found that type 1 diabetes was a risk factor for severe COVID-19 illness among US children. Additionally, evidence suggests that children with type 1 diabetes have been negatively affected during the pandemic.^22,23,24,25,26^ Our observation of type 1 diabetes as a risk factor for severe COVID-19 illness could be partially explained by complications from preexisting or new-onset diabetes in the setting of SARS-CoV-2 infection or indirect pandemic-related causes (eg, delays in seeking care, delays in diagnosis, and subsequent poor glycemic control among patients with type 1 diabetes).^22,23,26,27^

Anxiety and fear-related disorders, depressive disorders, and neurodevelopmental disorders (the latter driven by attention-deficit/hyperactivity disorder [ADHD] and autism spectrum disorders) were prevalent in the sample and were associated with a higher risk of hospitalization but not severe illness when hospitalized. Mental health disorders among US children are common, with ADHD (9.4%), anxiety (7.1%), and depression (3.2%) being the most commonly diagnosed.^28^ In addition, the COVID-19 pandemic has worsened the well-being of children and their parents,^29^ potentially because of ongoing social, health service, and academic disruptions (eg, loss of school-based mental health services, financial losses, food insecurity).^30^ It is unclear why children with mental health disorders might be hospitalized more frequently; it is possible that the effects of medications being used to treat the underlying condition are unknown or that clinicians might be more likely to hospitalize a child with a mental health disorder for closer observation or to provide additional support to the child or family.

Asthma was the most frequent diagnosed condition, significantly associated with risk of hospitalization. Asthma was not found to be associated with a higher risk of COVID-19 illness among hospitalized children, except among those aged 12 to 18 years. A previous analysis of 454 patients younger than 21 years at Children’s Hospital Colorado found asthma to be a risk factor for hospitalization and respiratory support but not critical care.^7^ Current evidence suggesting asthma as a risk factor for severe COVID-19 illness among adults is mixed.^31^ Thus, the role of asthma in severity of infection for both children and adults remains unclear.

Obesity is a known risk factor for severe COVID-19 illness in adults,^32,33,34^ and this study provides evidence of obesity as a risk factor among children. Prior descriptive and case series studies showed obesity as more frequent in children with severe COVID-19 illness compared with the general population.^2,35,36^ Obesity can have numerous negative health impacts that could explain this higher risk, including chronic inflammation, impaired immunity, and underlying cardiopulmonary disease.^37^

Children with epilepsy were at higher risk of hospitalization and severe COVID-19 illness when hospitalized. Evidence regarding epilepsy or seizures and COVID-19 has been scarce, although a recent study found a higher risk of fatality among patients with epilepsy and probable or confirmed COVID-19.^38^

Medical complexity and its association with severe COVID-19 illness among children is not well understood. Prior studies defined medical complexity as technology dependence (eg, gastrostomy status),^7^ which we also found to be associated with severe COVD-19. Our study used the PMCA definition of medical complexity, ie, as presence of NC-CD or C-CD (the latter including the presence of malignant neoplasms, progressive conditions, or conditions affecting ≥2 body systems).^17^ We found that children with C-CD and children with NC-CD were at a higher risk of hospitalization and severe illness when hospitalized compared with children without chronic disease. Additionally, risk ratios were higher among children with C-CD compared with children with NC-CD. Our finding that levels of medical complexity represent a risk factor for severe COVID-19 illness identifies a previously unidentified higher risk population, not clearly described in prior literature.

More than half of our sample consisted of adolescents (aged 12-18 years), and thus our results are most reflective of that age group. Age stratified analyses showed that certain conditions (asthma, neurodevelopmental disorders) were more frequent among patients aged 2 years and older, whereas prematurity and cardiac and circulatory congenital anomalies were the most frequent and associated with the highest risk of COVID-19 illness among patients younger than 2 years.

Premature birth affects 1 in 10 infants in the US^39^ and is a risk factor for long-term adverse sequelae, including those affecting respiratory system.^40^ Our finding supported prior findings of an association between prematurity and hospital admission,^7^ and showed that prematurity was also a risk factor for severe COVID-19 illness among hospitalized patients younger than 2 years. Future epidemiological analyses could shed light on the mechanisms of association between prematurity and severe COVID-19 illness and treatment approaches for this group of patients.

The sensitivity analysis revealed 3 indeterminate conditions (ie, coagulation and hemorrhagic disorders, other nutritional and metabolic disorders, diseases of white blood cells) as both frequent and associated with at least 1 outcome indicating severe COVID-19 illness (eFigure 3 in the Supplement). These heterogeneous CCSR categories include a wide range of conditions, including underlying conditions and those that could appear in the course of COVID-19 illness.^41,42,43,44,45,46^ Inclusion of these factors, which were strongly associated with at least 1 outcome of severe COVID-19 illness, likely resulted in reduced magnitudes for the other covariates.^47^

The second sensitivity analysis, performed on a subset of 20 773 children with at least 1 encounter prior to their first COVID-19 visit, found differences in the strength of associations between certain frequent conditions and risk of severe illness. It is possible that underascertainment of certain conditions because of using only pre–COVID-19 encounters resulted in a bias of the effect estimates.

### Limitations

This study has limitations. First, using *ICD-10-CM* diagnostic codes to identify COVID-19 cases might result in misclassification, although COVID-19 codes in PHD-SR showed high sensitivity and specificity with SARS-CoV-2 test results.^48^ Second, this cross-sectional analysis could not determine causal relationships between the underlying conditions and severe COVID-19 illness. Third, using *ICD-10-CM* codes to identify underlying medical conditions may have led to their misclassification (eg, misclassification of diabetes type) or underestimation of their prevalence (eg, underdiagnosis of obesity).^49,50^ For example, 2.5% of children in the full sample and 10.2% of hospitalized children had an obesity diagnosis, compared with 18.5% nationally.^51^ Future studies could use measured body mass index to more accurately capture the prevalence of obesity among children with COVID-19 and their associated risk of severe illness. Fourth, hospitalization risk estimates could be biased if (1) coding of conditions differed by care setting or (2) a child’s hospital admission was driven by factors other than severity, such as parents’ or health care professionals’ fear of progressive severity. Fifth, we used payer type as the only available indicator of socioeconomic status, but confounding by socioeconomic indicators (such as access to care) is possible. Sixth, this analysis was limited to frequent chronic conditions, which may have caused us to omit acute or rare conditions that were risk factors for severe COVID-19 illness. Seventh, this analysis was completed among children with inpatient or ED encounters; hence, the results are not representative of all children with COVID-19. Future analyses that include a broader sample of children with COVID-19 are important to shed light on these associations.

## Conclusions

This cross-sectional analysis showed that children with type 1 diabetes, cardiac and circulatory congenital anomalies, obesity, essential hypertension, epilepsy, neuropsychiatric disorders, and asthma as well as children with chronic disease were at an increased risk of hospitalization or severe COVID-19 illness. Children aged 1 year or younger with prematurity were at an increased risk of severe COVID-19 illness. Public health prevention and vaccine prioritization efforts might consider the potential for severe COVID-19 illness among children with these underlying medical conditions and chronic disease. Health care practitioners can consider the potential need for cautious clinical management of children with these conditions and COVID-19. Further epidemiologic investigation could provide insight into the causal pathways underlying our findings and identify other factors that place children at increased risk of severe COVID-19 illness.

## Notes

Supplement. **eFigure 1.** Inclusion and Exclusion Criteria for Cohort Selection **eFigure 2.** Inclusion and Exclusion Criteria for Identifying a List of Underlying Medical Conditions **eTable 1.** Most Frequent Underlying Medical Conditions in the Sample, by Care Setting **eFigure 3.** Association Between Underlying Medical Conditions and Risk of Hospitalization or Severe Illness When Hospitalized in the Sample, Including Likely Underlying, Indeterminate, and Likely Acute CCSR Categories **eTable 2.** Characteristics of the pre–COVID-19 Sample: Patients With at Least 1 Encounter Prior to Their First COVID-19 Diagnosis During an Emergency Department or Inpatient Encounter **eTable 3.** Most Frequent Underlying Medical Conditions Documented Prior to First COVID-19 Encounter in the Sample **eFigure 4.** Association Between Underlying Medical Conditions Documented Prior to First COVID-19 Encounter and Risk of Hospitalization or Severe Illness When Hospitalized in the Sample

## References

[1] Tsankov BK, Allaire JM, Irvine MA, . Severe COVID-19 infection and pediatric comorbidities: a systematic review and meta-analysis. Int J Infect Dis. 2021;103:246-256. doi:10.1016/j.ijid.2020.11.163 33227520 PMC7679116

[2] Kim L, Whitaker M, O’Halloran A, ; COVID-NET Surveillance Team. Hospitalization rates and characteristics of children aged <18 years hospitalized with laboratory-confirmed COVID-19—COVID-NET, 14 states, March 1–July 25, 2020. MMWR Morb Mortal Wkly Rep. 2020;69(32):1081-1088. doi:10.15585/mmwr.mm6932e3 32790664 PMC7440125

[3] Shekerdemian LS, Mahmood NR, Wolfe KK, ; International COVID-19 PICU Collaborative. Characteristics and outcomes of children with coronavirus disease 2019 (COVID-19) infection admitted to US and Canadian pediatric intensive care units. JAMA Pediatr. 2020;174(9):868-873. doi:10.1001/jamapediatrics.2020.1948 32392288 PMC7489842

[4] Alsaied T, Aboulhosn JA, Cotts TB, . Coronavirus disease 2019 (COVID-19) pandemic implications in pediatric and adult congenital heart disease. J Am Heart Assoc. 2020;9(12):e017224. doi:10.1161/JAHA.120.017224 32441586 PMC7429046

[5] Bellino S, Punzo O, Rota MC, ; COVID-19 WORKING GROUP. COVID-19 disease severity risk factors for pediatric patients in Italy. Pediatrics. 2020;146(4):e2020009399. doi:10.1542/peds.2020-009399 32665373

[6] Götzinger F, Santiago-García B, Noguera-Julián A, ; ptbnet COVID-19 Study Group. COVID-19 in children and adolescents in Europe: a multinational, multicentre cohort study. Lancet Child Adolesc Health. 2020;4(9):653-661. doi:10.1016/S2352-4642(20)30177-2 32593339 PMC7316447

[7] Graff K, Smith C, Silveira L, . Risk factors for severe COVID-19 in children. Pediatr Infect Dis J. 2021;40(4):e137-e145. doi:10.1097/INF.0000000000003043 33538539

[8] Van Cleave J, Gortmaker SL, Perrin JM. Dynamics of obesity and chronic health conditions among children and youth. JAMA. 2010;303(7):623-630. doi:10.1001/jama.2010.104 20159870

[9] von Elm E, Altman DG, Egger M, Pocock SJ, Gøtzsche PC, Vandenbroucke JP; STROBE Initiative. The Strengthening the Reporting of Observational Studies in Epidemiology (STROBE) statement: guidelines for reporting observational studies. Ann Intern Med. 2007;147(8):573-577. doi:10.7326/0003-4819-147-8-200710160-00010 17938396

[10] Premier. Premier Healthcare Database (COVID-19): data that informs and performs. Published April 10, 2020. Accessed February 08, 2021. http://offers.premierinc.com/rs/381-NBB-525/images/PHD_COVID-19_White_Paper.pdf

[11] US Centers for Disease Control and Prevention. New *ICD-10-CM* code for the 2019 novel coronavirus (COVID-19). April 1, 2020. Accessed April 26, 2021. https://www.cdc.gov/nchs/data/icd/Announcement-New-ICD-code-for-coronavirus-3-18-2020.pdf

[12] US Centers for Disease Control and Prevention. COVID-19 racial and ethnic health disparities. December 10, 2020. Accessed May 11, 2021. https://www.cdc.gov/coronavirus/2019-ncov/community/health-equity/racial-ethnic-disparities/index.html

[13] Agency for Healthcare Research and Quality. Chronic Condition Indicator (CCI) for *ICD-10-CM* (beta version). Accessed April 26, 2021. https://www.hcup-us.ahrq.gov/toolssoftware/chronic_icd10/chronic_icd10.jsp

[14] Agency for Healthcare Research and Quality. Clinical Classifications Software Refined (CCSR). Published November 2020. Accessed April 26, 2021. https://www.hcup-us.ahrq.gov/toolssoftware/ccsr/ccs_refined.jsp

[15] Wells BJ, Lenoir KM, Wagenknecht LE, . Detection of diabetes status and type in youth using electronic health records: the SEARCH for Diabetes in Youth Study. Diabetes Care. 2020;43(10):2418-2425. doi:10.2337/dc20-0063 32737140 PMC7510036

[16] Simon TD, Haaland W, Hawley K, Lambka K, Mangione-Smith R. Development and validation of the Pediatric Medical Complexity Algorithm (PMCA) version 3.0. Acad Pediatr. 2018;18(5):577-580. doi:10.1016/j.acap.2018.02.010 29496546 PMC6035108

[17] Spiegelman D, Hertzmark E. Easy SAS calculations for risk or prevalence ratios and differences. Am J Epidemiol. 2005;162(3):199-200. doi:10.1093/aje/kwi188 15987728

[18] Zou G. A modified Poisson regression approach to prospective studies with binary data. Am J Epidemiol. 2004;159(7):702-706. doi:10.1093/aje/kwh090 15033648

[19] White H. A heteroskedasticity-consistent covariance matrix estimator and a direct test for heteroskedasticity. Econometrica. 1980;48(4):817-838. doi:10.2307/1912934

[20] Simpson M, Collins C, Nash DB, Panesar LE, Oster ME. Coronavirus disease 2019 infection in children with pre-existing heart disease. J Pediatr. 2020;227:302-307.e2. doi:10.1016/j.jpeds.2020.07.069 32730815 PMC7384421

[21] Barron E, Bakhai C, Kar P, . Associations of type 1 and type 2 diabetes with COVID-19-related mortality in England: a whole-population study. Lancet Diabetes Endocrinol. 2020;8(10):813-822. doi:10.1016/S2213-8587(20)30272-2 32798472 PMC7426088

[22] Rabbone I, Schiaffini R, Cherubini V, Maffeis C, Scaramuzza A; Diabetes Study Group of the Italian Society for Pediatric Endocrinology and Diabetes. Has COVID-19 delayed the diagnosis and worsened the presentation of type 1 diabetes in children? Diabetes Care. 2020;43(11):2870-2872. doi:10.2337/dc20-1321 32778554

[23] Kamrath C, Mönkemöller K, Biester T, . Ketoacidosis in children and adolescents with newly diagnosed type 1 diabetes during the COVID-19 pandemic in Germany. JAMA. 2020;324(8):801-804. doi:10.1001/jama.2020.13445 32702751 PMC7372511

[24] Cherubini V, Gohil A, Addala A, . Unintended consequences of coronavirus disease-2019: remember general pediatrics. J Pediatr. 2020;223:197-198. doi:10.1016/j.jpeds.2020.05.004 32437758 PMC7207102

[25] Basatemur E, Jones A, Peters M, Ramnarayan P. Paediatric critical care referrals of children with diabetic ketoacidosis during the COVID-19 pandemic. Arch Dis Child. 2021;106(4):e21. doi:10.1136/archdischild-2020-320471 32938625

[26] Verma A, Rajput R, Verma S, Balania VKB, Jangra B. Impact of lockdown in COVID 19 on glycemic control in patients with type 1 diabetes mellitus. Diabetes Metab Syndr. 2020;14(5):1213-1216. doi:10.1016/j.dsx.2020.07.016 32679527 PMC7357511

[27] Singh AK, Singh R. Hyperglycemia without diabetes and new-onset diabetes are both associated with poorer outcomes in COVID-19. Diabetes Res Clin Pract. 2020;167:108382. doi:10.1016/j.diabres.2020.108382 32853686 PMC7445123

[28] National Center on Birth Defects and Developmental Disabilities, Centers for Disease Control and Prevention. Data and statistics on children's mental health. Accessed November 11, 2020. https://www.cdc.gov/childrensmentalhealth/data.html

[29] Patrick SW, Henkhaus LE, Zickafoose JS, . Well-being of parents and children during the COVID-19 pandemic: a national survey. Pediatrics. 2020;146(4):e2020016824. doi:10.1542/peds.2020-016824 32709738

[30] Leeb RT, Bitsko RH, Radhakrishnan L, Martinez P, Njai R, Holland KM. Mental health-related emergency department visits among children aged <18 years during the COVID-19 pandemic—United States, January 1-October 17, 2020. MMWR Morb Mortal Wkly Rep. 2020;69(45):1675-1680. doi:10.15585/mmwr.mm6945a3 33180751 PMC7660659

[31] US Centers for Disease Control and Prevention. Evidence used to update the list of underlying medical conditions that increase a person’s risk of severe illness from COVID-19. Updated March 29, 2021. Accessed April 26, 2021. https://www.cdc.gov/coronavirus/2019-ncov/need-extra-precautions/evidence-table.html

[32] Chang TH, Chou CC, Chang LY. Effect of obesity and body mass index on coronavirus disease 2019 severity: a systematic review and meta-analysis. Obes Rev. 2020;21(11):e13089. doi:10.1111/obr.13089 32929833

[33] Pranata R, Lim MA, Yonas E, . Body mass index and outcome in patients with COVID-19: a dose-response meta-analysis. Diabetes Metab. 2021;47(2):101178. doi:10.1016/j.diabet.2020.07.005 32738402 PMC7388778

[34] Tartof SY, Qian L, Hong V, . Obesity and mortality among patients diagnosed with COVID-19: results from an integrated health care organization. Ann Intern Med. 2020;173(10):773-781. doi:10.7326/M20-3742 32783686 PMC7429998

[35] Bixler D, Miller AD, Mattison CP, ; Pediatric Mortality Investigation Team. SARS-CoV-2-associated deaths among persons aged <21 years—United States, February 12-July 31, 2020. MMWR Morb Mortal Wkly Rep. 2020;69(37):1324-1329. doi:10.15585/mmwr.mm6937e4 32941417

[36] Zachariah P, Johnson CL, Halabi KC, ; Columbia Pediatric COVID-19 Management Group. Epidemiology, clinical features, and disease severity in patients with coronavirus disease 2019 (COVID-19) in a children’s hospital in New York City, New York. JAMA Pediatr. 2020;174(10):e202430. doi:10.1001/jamapediatrics.2020.2430 32492092 PMC7270880

[37] Nogueira-de-Almeida CA, Del Ciampo LA, Ferraz IS, Del Ciampo IRL, Contini AA, Ued FDV. COVID-19 and obesity in childhood and adolescence: a clinical review. J Pediatr (Rio J). 2020;96(5):546-558. doi:10.1016/j.jped.2020.07.001 32768388 PMC7402231

[38] Cabezudo-García P, Ciano-Petersen NL, Mena-Vázquez N, Pons-Pons G, Castro-Sánchez MV, Serrano-Castro PJ. Incidence and case fatality rate of COVID-19 in patients with active epilepsy. Neurology. 2020;95(10):e1417-e1425. doi:10.1212/WNL.0000000000010033 32554773

[39] Martin JA, Hamilton BE, Osterman MJK. Births in the United States, 2018. NCHS Data Brief. 2019;(346):1-8. 31442195

[40] Saigal S, Doyle LW. An overview of mortality and sequelae of preterm birth from infancy to adulthood. Lancet. 2008;371(9608):261-269. doi:10.1016/S0140-6736(08)60136-1 18207020

[41] Connors JM, Levy JH. COVID-19 and its implications for thrombosis and anticoagulation. Blood. 2020;135(23):2033-2040. doi:10.1182/blood.2020006000 32339221 PMC7273827

[42] Goldhaber SZ. Risk factors for venous thromboembolism. J Am Coll Cardiol. 2010;56(1):1-7. doi:10.1016/j.jacc.2010.01.057 20620709

[43] Di Filippo L, Formenti AM, Rovere-Querini P, . Hypocalcemia is highly prevalent and predicts hospitalization in patients with COVID-19. Endocrine. 2020;68(3):475-478. doi:10.1007/s12020-020-02383-5 32533508 PMC7292572

[44] Liu J, Han P, Wu J, Gong J, Tian D. Prevalence and predictive value of hypocalcemia in severe COVID-19 patients. J Infect Public Health. 2020;13(9):1224-1228. doi:10.1016/j.jiph.2020.05.029 32622796 PMC7306733

[45] Zhao Q, Meng M, Kumar R, . Lymphopenia is associated with severe coronavirus disease 2019 (COVID-19) infections: a systemic review and meta-analysis. Int J Infect Dis. 2020;96:131-135. doi:10.1016/j.ijid.2020.04.086 32376308 PMC7196544

[46] Huang G, Kovalic AJ, Graber CJ. Prognostic value of leukocytosis and lymphopenia for coronavirus disease severity. Emerg Infect Dis. 2020;26(8):1839-1841. doi:10.3201/eid2608.201160 32384045 PMC7392413

[47] Arah OA. The role of causal reasoning in understanding Simpson’s paradox, Lord’s paradox, and the suppression effect: covariate selection in the analysis of observational studies. Emerg Themes Epidemiol. 2008;5:5. doi:10.1186/1742-7622-5-5 18302750 PMC2266743

[48] Kadri SS, Gundrum J, Warner S, . Uptake and accuracy of the diagnosis code for COVID-19 among US hospitalizations. JAMA. 2020;324(24):2553-2554. doi:10.1001/jama.2020.20323 33351033 PMC7756233

[49] Sharifi M, Rifas-Shiman SL, Marshall R, . Evaluating the implementation of expert committee recommendations for obesity assessment. Clin Pediatr (Phila). 2013;52(2):131-138. doi:10.1177/0009922812471712 23378479

[50] Borgmeyer A, Ercole PM, Niesen A, Strunk RC. Lack of recognition, diagnosis, and treatment of overweight/obesity in children hospitalized for asthma. Hosp Pediatr. 2016;6(11):667-676. doi:10.1542/hpeds.2015-0242 27733428

[51] Hales CM, Carroll MD, Fryar CD, Ogden CL. Prevalence of obesity among adults and youth: United States, 2015-2016. NCHS Data Brief. 2017;(288):1-8. 29155689

[52] Lovinsky-Desir S, Deshpande DR, De A, . Asthma among hospitalized patients with COVID-19 and related outcomes. J Allergy Clin Immunol. 2020;146(5):1027-1034.e4. doi:10.1016/j.jaci.2020.07.026 32771560 PMC7409831

[53] Debevec T, Burtscher J, Millet GP. Preterm birth: potential risk factor for greater COVID-19 severity? Respir Physiol Neurobiol. 2020;280:103484. doi:10.1016/j.resp.2020.103484 32599161 PMC7319613

