# Supplementary material for: Underlying Medical Conditions Associated With Severe COVID-19 Illness Among Children
Source: JAMA Netw Open. 2021 Jun 7;4(6):e2111182. doi: 10.1001/jamanetworkopen.2021.11182 (PMC8185607; doi:10.1001/jamanetworkopen.2021.11182)
Supplement: Supplement. — eFigure 1. Inclusion and Exclusion Criteria for Cohort Selection eFigure 2. Inclusion and Exclusion Criteria for Identifying a List of Underlying Medical Conditions eTable 1. Most Frequent Underlying Medical Conditions in the Sample, by Care Setting eFigure 3. Association Between Underlying Medical Conditions and Risk of Hospitalization or Severe Illness When Hospitalized in the Sample, Including Likely Underlying, Indeterminate, and Likely Acute CCSR Categories eTable 2. Characteristics of the pre–COVID-19 Sample: Patients With at Least 1 Encounter Prior to Their First COVID-19 Diagnosis During an Emergency Department or Inpatient Encounter eTable 3. Most Frequent Underlying Medical Conditions Documented Prior to First COVID-19 Encounter in the Sample eFigure 4. Association Between Underlying Medical Conditions Documented Prior to First COVID-19 Encounter and Risk of Hospitalization or Severe Illness When Hospitalized in the Sample [file jamanetwopen-e2111182-s001.pdf]

Supplemental Online Content

Kompaniyets L, Agathis NT, Nelson JM, et al. Underlying medical conditions associated with severe COVID-19 illness among children. *JAMA Netw Open*. 2021;4(6):e2111182.
doi:10.1001/jamanetworkopen.2021.11182

eFigure 1. Inclusion and Exclusion Criteria for Cohort Selection

eFigure 2. Inclusion and Exclusion Criteria for Identifying a List of Underlying Medical Conditions

eTable 1. Most Frequent Underlying Medical Conditions in the Sample, by Care Setting

eFigure 3. Association Between Underlying Medical Conditions and Risk of Hospitalization or Severe Illness When Hospitalized in the Sample, Including Likely Underlying, Indeterminate, and Likely Acute CCSR Categories

eTable 2. Characteristics of the pre–COVID-19 Sample: Patients With at Least 1 Encounter Prior to Their First COVID-19 Diagnosis During an Emergency Department or Inpatient Encounter

eTable 3. Most Frequent Underlying Medical Conditions Documented Prior to First COVID-19 Encounter in the Sample

eFigure 4. Association Between Underlying Medical Conditions Documented Prior to First COVID-19 Encounter and Risk of Hospitalization or Severe Illness When Hospitalized in the Sample

This supplemental material has been provided by the authors to give readers additional information about their work.

© 2021 Kompaniyets L et al. *JAMA Network Open.*

eFigure 1. Inclusion and Exclusion Criteria for Cohort Selection

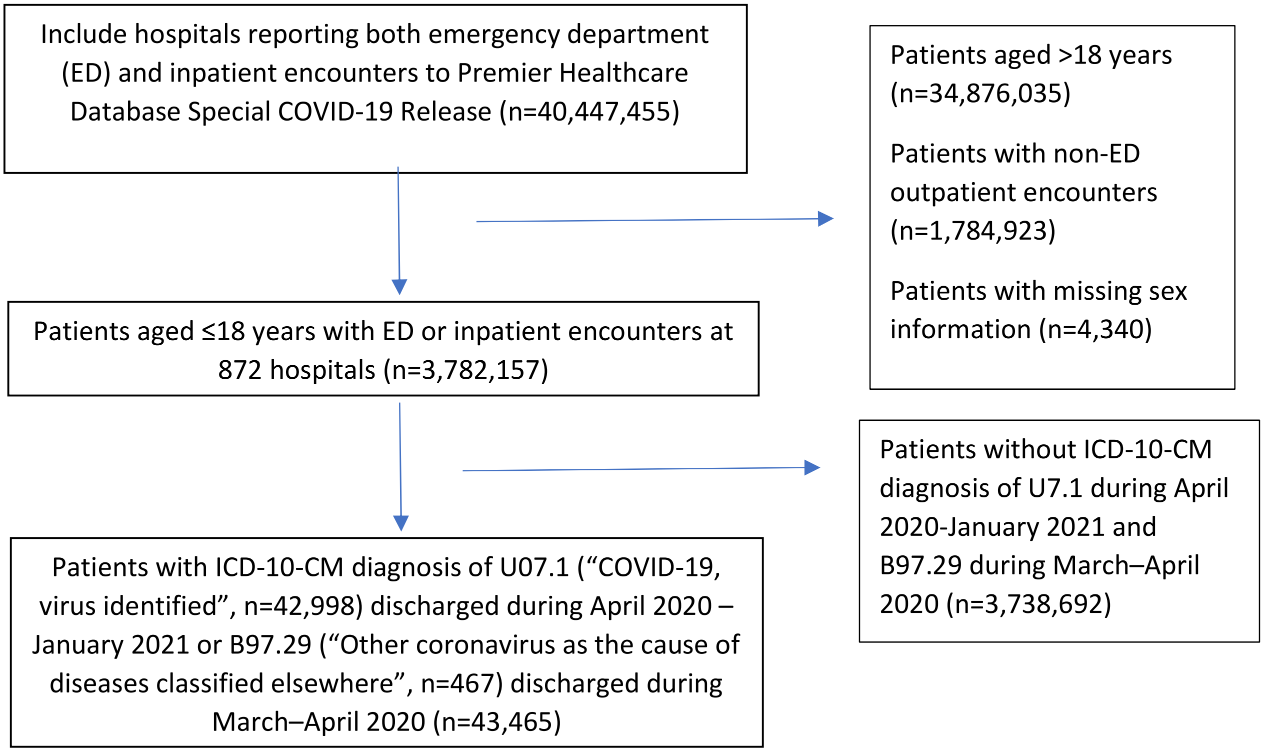

© 2021 Kompaniyets L et al. *JAMA Network Open.*

eFigure 2. Inclusion and Exclusion Criteria for Identifying a List of Underlying Medical Conditions

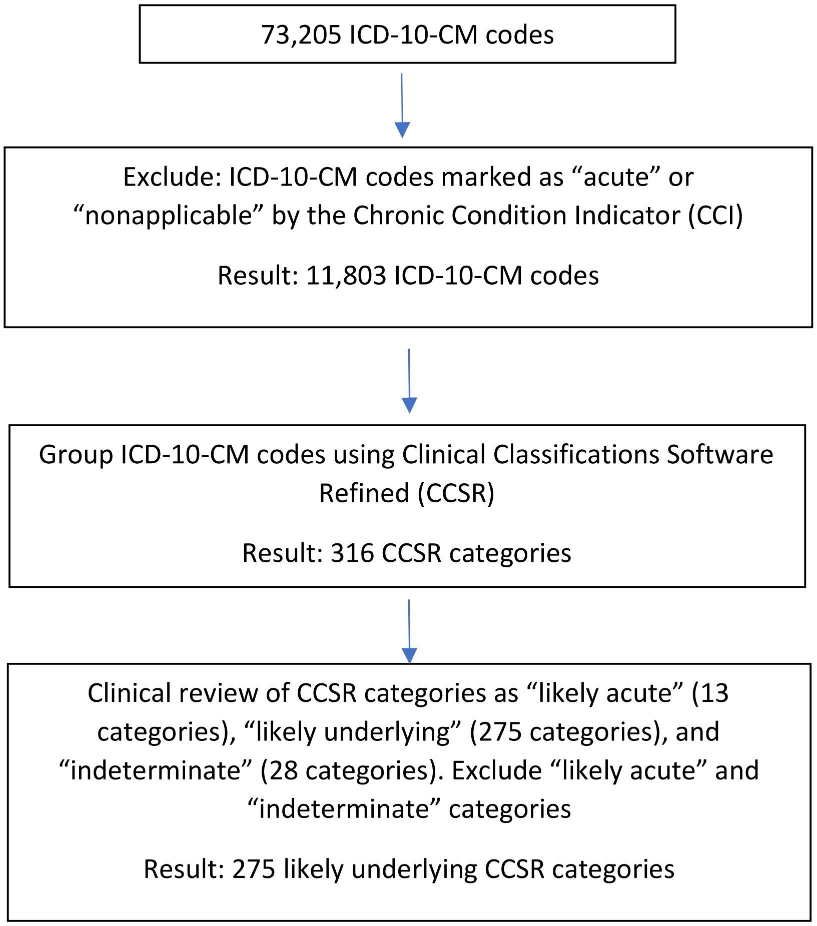

© 2021 Kompaniyets L et al. *JAMA Network Open.*

eTable 1. Most Frequent Underlying Medical Conditions in the Sample, by Care Setting

**ED subset: Children with a COVID-19 ED encounter only**	**n**	**%**	**Inpatient subset: Children with a COVID-19 inpatient encounter**	**n**	**%**
Asthma	3,809	9.7	Asthma	607	14.1
Neurodevelopmental disorders	1,236	3.2	Neurodevelopmental disorders	454	10.6
Anxiety and fear-related disorders	1,001	2.6	Obesity	437	10.2
Depressive disorders	859	2.2	Anxiety and fear-related disorders	373	8.7
Obesity	634	1.6	Depressive disorders	350	8.1
Esophageal disorders	612	1.6	Esophageal disorders	267	6.2
Tobacco-related disorders	580	1.5	Epilepsy; convulsions	235	5.5
Other upper respiratory disease	413	1.1	Other specified status	233	5.4
Epilepsy; convulsions	324	0.8	Cardiac and circulatory congenital anomalies	230	5.3
Headache; including migraine	297	0.8	Essential hypertension	211	4.9
Other specified and unspecified congenital anomalies	290	0.7	Trauma- and stressor-related disorders	187	4.3
Cardiac and circulatory congenital anomalies	280	0.7	Type 1 diabetes	154	3.6
Menstrual disorders	262	0.7	Sleep wake disorders	149	3.5
Trauma- and stressor-related disorders	245	0.6	Malnutrition	144	3.3
Essential hypertension	225	0.6	Aplastic anemia	130	3.0
Type 2 diabetes	215	0.6	Implant device or graft-related encounter	117	2.7
Bipolar and related disorders	200	0.5	Type 2 diabetes	105	2.4

[a] Underlying medical conditions were defined by (1) using Chronic Condition Indicator to identify chronic ICD-10-CM codes, (2) aggregating the codes into a smaller number of meaningful categories using Clinical Classifications Software Refined (CCSR), (3) a clinical review of CCSR categories that classified the CCSR codes as “likely underlying”, “indeterminate” and “likely acute”, (4) including only “likely underlying” CCSR categories.
[b] The sample is defined as children ≤18 years with a COVID-19 diagnosis during an emergency department (ED) or inpatient encounter in hospitals that report both emergency department and inpatient encounters to Premier Healthcare Database Special COVID-19 Release, March 2020–January 2021.

© 2021 Kompaniyets L et al. *JAMA Network Open.*

eFigure 3. Association Between Underlying Medical Conditions[a] and Risk of Hospitalization or Severe Illness When Hospitalized in the Sample,[b,c] Including Likely Underlying, Indeterminate, and Likely Acute CCSR Categories

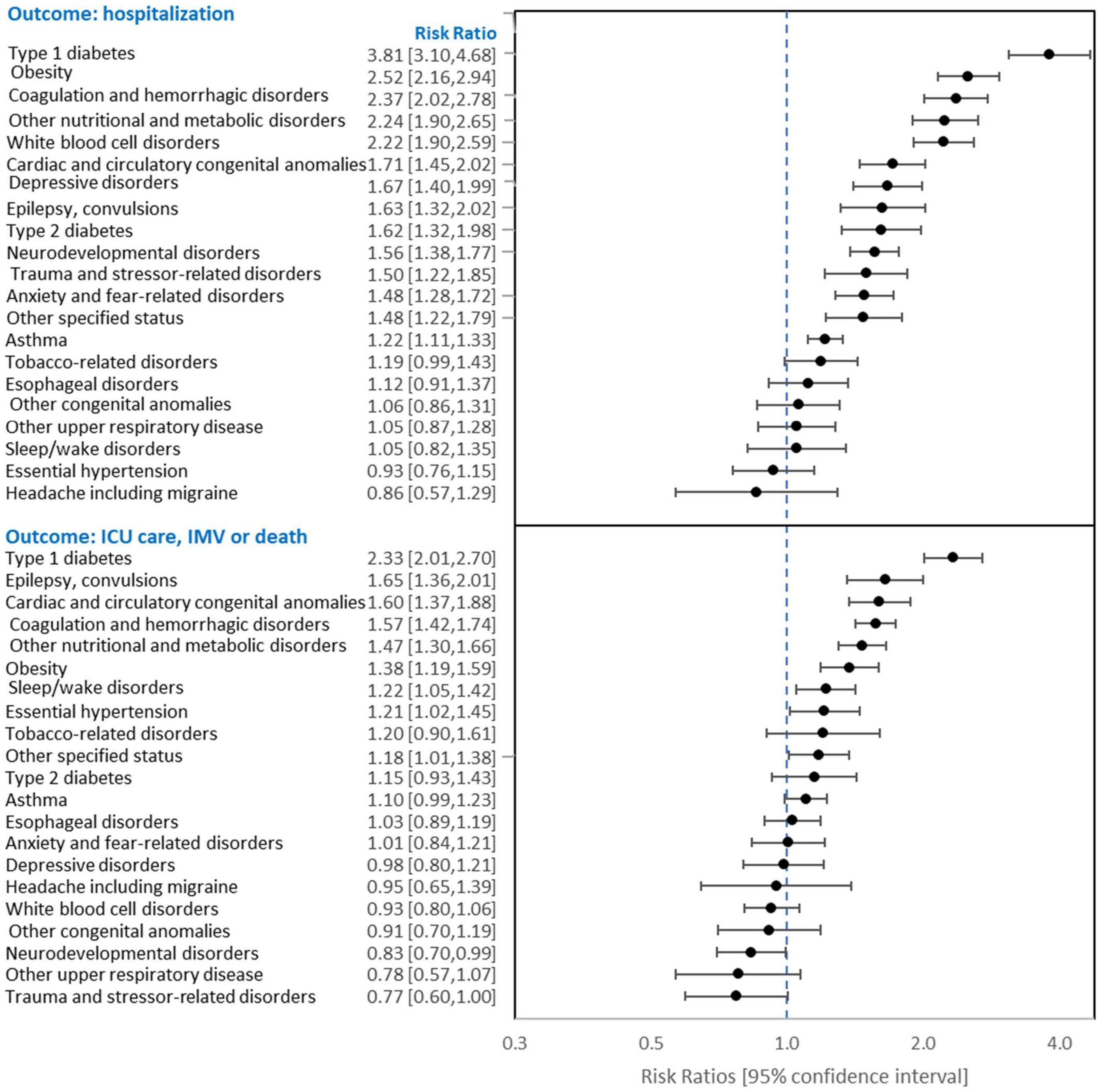

Note: Abbreviations: ICU — intensive care unit; IMV— invasive mechanical ventilation.

[a] Underlying medical conditions were defined by (1) using Chronic Condition Indicator to identify chronic ICD-10-CM codes; (2) aggregating the codes into a smaller number of meaningful categories using Clinical Classifications Software Refined (CCSR); (3) a clinical review of CCSR categories that classified the CCSR codes as “likely underlying”, “indeterminate” and “likely acute”

[b] The sample is defined as children ≤18 years with a COVID-19 diagnosis during an emergency department (ED) or inpatient encounter in hospitals that report both emergency department and inpatient encounters to Premier Healthcare Database Special COVID-19 Release, March 2020–January 2021

[c]The figure includes results of two generalized linear models with Poisson distribution that include all underlying medical conditions of interest, age group, sex, race/ethnicity, payer type, urbanicity, Census region, admission month, admission month squared. Reference group for each underlying condition was absence of that condition; reference group for type 1 and type 2 diabetes was no diabetes. Single dots on the graph represent adjusted risk ratios, confidence intervals are shown by error bars, and the dotted vertical line represents the null (risk ratio of 1.0).

© 2021 Kompaniyets L et al. *JAMA Network Open.*

eTable 2. Characteristics of the pre–COVID-19 Sample[a]: Patients With at Least 1 Encounter Prior to Their First COVID-19 Diagnosis During an Emergency Department or Inpatient Encounter

Characteristic[b]	Full sample: Children with an ED or inpatient COVID-19 encounter and at least 1 pre-COVID encounter n =20,773		ED subset: Children with an ED encounter only n=18,850 (90.7%)		Inpatient subset: Children with an inpatient encounter n=1,923 (9.3%)		Inpatient subset: Children with no severe illness n=1,371		Inpatient subset: Children with severe illness n=552	
	N	%	N	%	N	%	N	%	N	%
	20,773	100.0	18,850	100.0	1,923	100.0	1,371	100.0	552	100.0
With underlying medical conditions[c](CCI/CCSR/clin. review)	7,601	36.6	6,465	34.3	1,136	59.1	737	53.8	399	72.3
With chronic disease [d] (PMCA)	5,105	24.6	4,177	22.2	928	48.3	573	41.8	355	64.3
Non-complex chronic	3,346	16.1	3,014	16.0	332	17.3	216	15.8	116	21.0
Complex chronic	1,759	8.5	1,163	6.2	596	31.0	357	26.0	239	43.3
Sex										
Female	11,132	53.6	10,046	53.3	1,086	56.5	824	60.1	262	47.5
Male	9,641	46.4	8,804	46.7	837	43.5	547	39.9	290	52.5
Age, years										
<1	3,072	14.8	2,685	14.2	387	20.1	292	21.3	95	17.2
1	1,625	7.8	1,518	8.1	107	5.6	82	6.0	25	4.5
2–5	2,749	13.2	2,531	13.4	218	11.3	137	10.0	81	14.7
6–11	3,328	16.0	3,123	16.6	205	10.7	119	8.7	86	15.6
12–18	9,999	48.1	8,993	47.7	1,006	52.3	741	54.0	265	48.0

[a] The sample is defined as children ≤18 years with at least one encounter prior to their first COVID-19 diagnosis during an emergency department (ED) or inpatient encounter, in hospitals that report both emergency department and inpatient encounters to Premier Healthcare Database, March–November 2020
[b] Some categories may not add up to 100%, due to rounding or missing/unknown values or cell size suppression (<10)
[c]Underlying medical conditions were defined by (1) using Chronic Condition Indicator to identify chronic ICD-10-CM codes in encounters prior to the first COVID-19 encounter; (2) aggregating the codes into a smaller number of meaningful categories using Clinical Classifications Software Refined (CCSR).
[d] Chronic disease was defined by the Pediatric Medical Complexity Algorithm (PMCA)

© 2021 Kompaniyets L et al. *JAMA Network Open.*

eTable 3. Most Frequent Underlying Medical Conditions[a] Documented Prior to First COVID-19 Encounter in the Sample[b]

**Full sample: Children with a COVID-19 ED or inpatient encounter**	**n**	**%**
Asthma	2,178	10.5
Neurodevelopmental disorders	995	4.8
Anxiety and fear-related disorders	754	3.6
Depressive disorders	669	3.2
Esophageal disorders	650	3.1
Obesity	498	2.4
Other specified and unspecified upper respiratory disease	376	1.8
Cardiac and circulatory congenital anomalies	356	1.7
Epilepsy; convulsions	354	1.7
Other specified and unspecified congenital anomalies	342	1.6
Other specified status	328	1.6
Diseases of white blood cells	299	1.4
Tobacco-related disorders	295	1.4
Menstrual disorders	281	1.4
Other specified and unspecified nutritional and metabolic disorders	280	1.3
Headache; including migraine	268	1.3
Trauma- and stressor-related disorders	247	1.2
Sleep wake disorders	240	1.2
Musculoskeletal congenital conditions	234	1.1
Essential hypertension	224	1.1
Implant device or graft related encounter	221	1.1
Genitourinary congenital anomalies	198	1.0
Diabetes	297	1.5
Type 2 diabetes	155	0.8
Type 1 diabetes	142	0.7

[a]Underlying medical conditions were defined by (1) using Chronic Condition Indicator to identify chronic ICD-10-CM codes in encounters prior to the first COVID-19 encounter; (2) aggregating the codes into a smaller number of meaningful categories using Clinical Classifications Software Refined (CCSR).
[b] The sample is defined as children ≤18 years with at least one encounter prior to their first COVID-19 diagnosis during an emergency department (ED) or inpatient encounter, in hospitals that report both emergency department and inpatient encounters to Premier Healthcare Database Special COVID-19 Release, March 2020–January 2021

© 2021 Kompaniyets L et al. *JAMA Network Open.*

eFigure 4. Association Between Underlying Medical Conditions[a] Documented Prior to First COVID-19 Encounter and Risk of Hospitalization or Severe Illness When Hospitalized in the Sample[b,c]

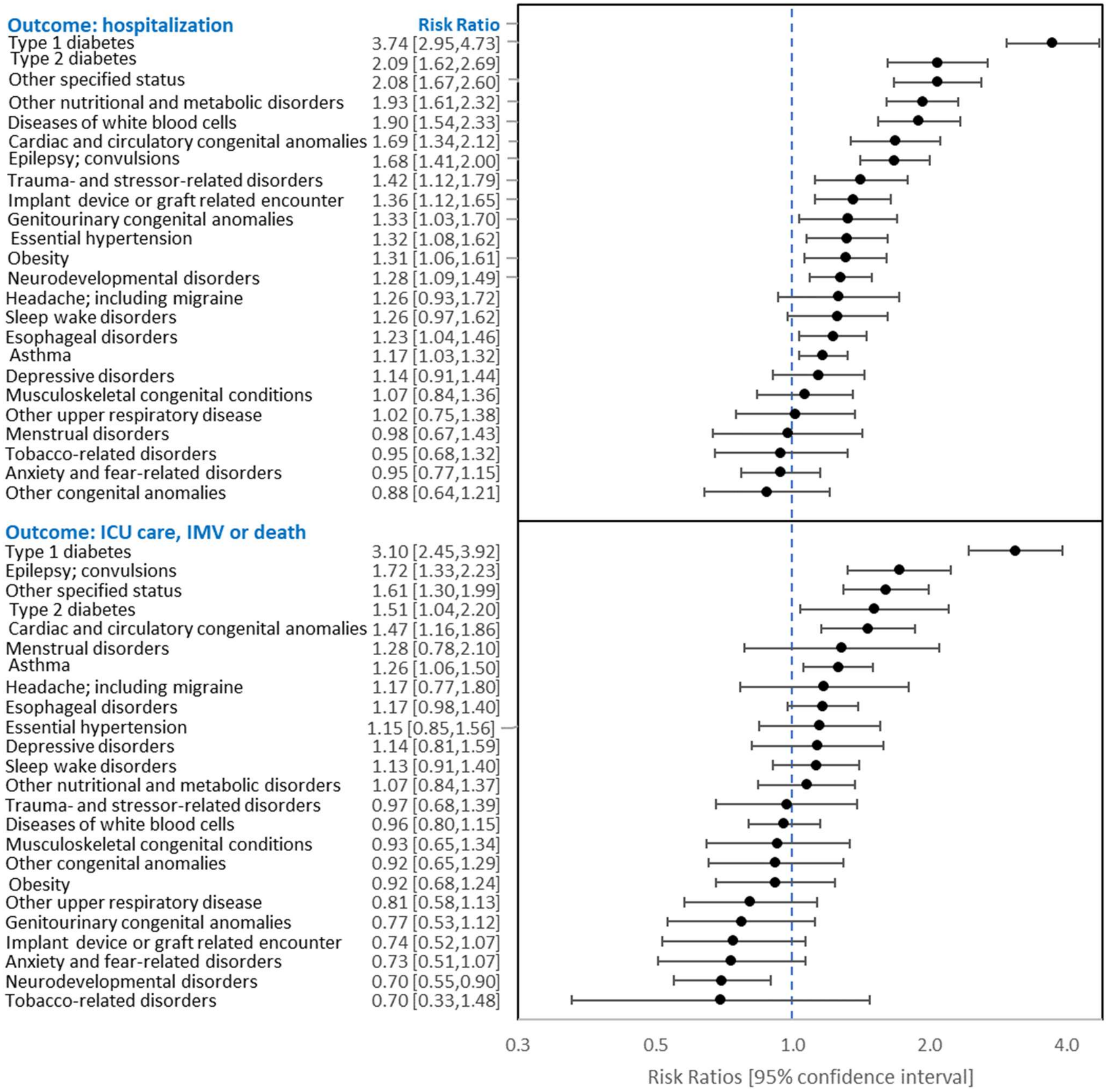

Note: Abbreviations: ICU — intensive care unit; IMV— invasive mechanical ventilation

[a] Underlying medical conditions were defined by (1) using Chronic Condition Indicator to identify chronic ICD-10-CM codes in encounters prior to the first COVID-19 encounter; (2) aggregating the codes into a smaller number of meaningful categories using Clinical Classifications Software Refined (CCSR).

[b] The sample is defined as children ≤18 years with at least one encounter prior to their first COVID-19 diagnosis during an emergency department (ED) or inpatient encounter, in hospitals that report both emergency department and inpatient encounters to Premier Healthcare Database Special COVID-19 Release, March 2020–January 2021. Children with unknown sex were omitted from the sample, as they predicted the outcome perfectly.

[c] The figure includes the results of two generalized linear models with Poisson distribution that include all underlying medical conditions of interest, age group, sex, race/ethnicity, payer type, urbanicity, Census region, admission month, admission month squared. Reference group for each underlying condition was absence of that condition; reference group for type 1 and type 2 diabetes was no diabetes. Single dots on the graph represent adjusted risk ratios, confidence intervals are shown by error bars, and the dotted vertical line represents the null (risk ratio of 1.0).

© 2021 Kompaniyets L et al. *JAMA Network Open.*
